# Use of estimands in cluster randomised trials: A review

**DOI:** 10.1177/17407745251415538

**Published:** 2026-02-17

**Authors:** Dongquan Bi, Andrew Copas, Brennan C Kahan

**Affiliations:** 1 MRC Clinical Trials Unit at UCL, Institute of Clinical Trials and Methodology, UCL, London, UK

**Keywords:** Estimand, cluster randomised trial, intercurrent event, ICH E9(R1)

## Abstract

**Background:**

An estimand is a clear description of the treatment effect a study aims to quantify. The ICH E9(R1) addendum lists five attributes that should be described as part of the estimand definition. However, the addendum was primarily developed for individually randomised trials. Cluster randomised trials, in which groups of individuals are randomised, have additional considerations for defining estimands (e.g. how individuals and clusters are weighted, how cluster-level intercurrent events are handled). However, it is currently unknown if estimands are being used in cluster randomised trials, or whether the considerations specific to cluster randomised trials are being described.

**Methods:**

We reviewed 73 cluster randomised trials published between October 2023 and January 2024 that were indexed in MEDLINE. For each trial, we assessed whether the estimand for the primary outcome was described, or if not, whether it could be inferred from the statistical methods. We also assessed whether considerations specific to cluster randomised trials were described or inferable, how trials were analysed and whether key assumptions being made in the analysis (e.g. ‘no informative cluster size’) could be identified.

**Results:**

No trials attempted to describe the estimand for their primary outcome. We were able to infer the five attributes outlined in ICH E9(R1) in only 49% of trials, and when including additional considerations specific to cluster randomised trials, this figure dropped to 21%. Key drivers of this ambiguity were lack of clarity around whether individual- or cluster-average effects were of interest (unclear in 63% of trials), and how cluster-level intercurrent events were handled (unclear in 21% of trials for which this was applicable). Over half of trials used mixed-effects models or generalising estimating equations with an exchangeable correlation structure, which make the assumption that there is no informative cluster size; however, only one of these trials performed sensitivity analyses to evaluate robustness of results to deviations from this assumption. There were 14% of trials that used independence estimating equations or the analysis of cluster-level summaries; however, because no trials stated whether they were targeting the individual- or cluster-average effect, it was impossible to determine whether these methods implemented the appropriate weighting scheme and were thus unbiased.

**Conclusion:**

The uptake of estimands in published cluster randomised trial articles is low, making it difficult to ascertain which questions were being investigated or whether statistical estimators were appropriate for those questions. This highlights an urgent need to develop guidelines on defining estimands that cover unique aspects of cluster randomised trials to ensure clarity of research questions in these trials.

## Introduction

An estimand is a clear description of the treatment effect a study aims to quantify.^1,2^ Estimands can help clarify the question a study sets out to investigate and help trial investigators to choose appropriate statistical methods to answer their target question. In 2019, the ICH E9(R1) addendum introduced a structured approach to defining estimands, comprising the specification of five attributes: (1) population of patients, (2) treatment conditions being compared, (3) the endpoint, (4) population-level summary measure and (5) how intercurrent events (post-randomisation events that affect interpretation or existence of outcomes, such as treatment non-adherence) are handled.^1^

However, the ICH E9(R1) addendum was developed by medicines regulators in conjunction with the pharmaceutical industry, and as such was primarily developed with individually randomised trials in mind. Cluster randomised trials (CRTs), where groups of individuals are randomised, may require specification of additional attributes. For example, investigators also need to consider the population of clusters for which they wish to estimate the treatment effect;^3–7^ how individuals and clusters are weighted (e.g. individual- vs cluster-average effect);^5,7–12^ whether marginal or cluster-specific effects are of interest;^5,11,13,14^ and how intercurrent events that occur at the cluster level (such as if a cluster decided not to implement the intervention) are handled.^5^ Table 1 provides a summary of some additional considerations.

**Table 1.** Summary of some additional considerations for defining estimands in cluster randomised trials.

Item	Description
Population of clusters	Clusters that are targeted by the research question
How individuals or clusters are weighted in the estimand (e.g. individual- or cluster-average treatment effect)	Individuals and clusters can be weighted differently in the estimand, e.g. by assigning equal weight to each individual (individual-average effect) or to each cluster (cluster-average effect) (see Appendix 1 for further explanation)
Marginal or cluster-specific treatment effect	Potential outcomes can be summarised and contrasted in different ways, e.g. a marginal effect summarises potential outcomes across all individuals for each treatment condition before contrasting, while a cluster-specific effect summarises and contrasts within each cluster first then averages across clusters
Strategies for handling cluster-level intercurrent events	Intercurrent events could occur at the level of cluster, e.g. if clusters did not implement or discontinued their assigned treatment. The same strategies that can be used to handle individual-level intercurrent events could be used to handle cluster-level intercurrent events

Failure to take these additional considerations into account when defining estimands for CRTs can both create ambiguity around trial objectives and hamper appraisal around the choice of estimator. For instance, common estimators for CRTs include generalising estimating equations (GEEs), mixed-effects models, independence estimating equations (IEEs) and the analysis of cluster-level summaries. Each estimator requires certain assumptions in order to be unbiased for a specific estimand. For instance, when there is informative cluster size (i.e. when outcomes or treatment effects vary according to cluster size), IEEs and the analysis of cluster-level summaries will only be unbiased if an appropriate weighting scheme is used which is aligned to the target estimand (e.g. individual- or cluster-average).^9^ Furthermore, under informative cluster size, both GEEs with an exchangeable correlation structure (termed ‘GEEs (exch)’ hereafter) and mixed-effect models may be biased for both the individual- and cluster-average effects.^8,15–17^ Without precise definition of the estimand including aspects unique to CRTs, it is impossible to know whether a trial’s estimator is aligned to its overall objective, or what assumptions are being made.

Despite growing recognition around the importance of estimands, current reporting guidelines for CRTs were established before the introduction of the ICH E9(R1) addendum,^7^ and it is currently unclear if estimands are being used in CRTs. Furthermore, due to lack of specific guidance on defining estimands in CRTs, even if estimands are being used, it is unclear whether studies are incorporating the considerations specific to CRTs. We therefore undertook a review of published CRTs to determine how often estimands are used, whether considerations specific to CRTs are being described and whether the key assumptions of the chosen estimators could be identified and evaluated.

## Method

### Search strategy

We searched on 23 January 2024 for articles reporting results from a CRT published between 1 October 2023 and 15 January 2024 on MEDLINE. This time period was selected based on an initial exploratory search which indicated that we could expect around 200 records to screen across this period. We aimed for around 200 articles to screen under the assumption that at least a quarter (n = 50) would be eligible to be included in the full text review, which is in line with previous research on the use of estimands in individually randomised trials.^18^ The full search strategy is available in Appendix 2 in the Supplemental Material. Briefly, the search strategy contained terms to (1) select a randomised controlled design, such as with publication type of ‘randomised controlled trial’ or with keyword ‘random’ in the abstract; (2) select a cluster design, such as with the MeSH term ‘cluster random’; and (3) exclude ineligible articles (described below), such as with publication type of ‘review’ or with keyword ‘protocol’ in the title or abstract.

### Eligibility

Parallel-group CRTs were eligible with no restrictions on medical conditions or type of intervention. Crossover, stepped wedge and factorial designs were excluded owing to additional considerations in defining estimands and different statistical considerations in these trials. Other exclusion criteria were pilot or feasibility studies, non-randomised studies, secondary analyses or a follow-up of a previously published trial, CRTs with cost-effectiveness as the primary outcome, articles with more than one trial reported, meta-analyses, systematic reviews, interim analyses and letters to the editor or commentaries.

Title and abstract screening for eligibility was performed by a single reviewer (D.B.). Queries regarding eligibility were discussed with at least one other author (B.C.K. and/or A.C.).

### Data extraction

Data were extracted using a piloted standardised extraction form. The extraction form was built on the Qualtrics Platform. The full extraction form is available in Appendix 3 in the Supplemental Material. One author (D.B.) extracted data for all eligible articles. Another author (B.C.K.) independently checked all extractions against the source article; discrepancies were resolved by discussion.

Extracted data included trial characteristics, whether the estimand was described, and if not, whether it could be inferred from the statistical methods, the types of intercurrent events that occurred, the type of estimand that was used and the statistical methods. Data on the estimand and statistical methods were extracted for the primary estimand for the trial’s primary outcome. Rules for determining the trial’s primary outcome and primary estimand are given in Appendix 4 in the Supplemental Material.

We evaluated whether certain types of intercurrent events were applicable. An intercurrent event was deemed applicable if the manuscript indicated it had occurred during the trial, or was considered during the trial’s planning stages, for instance, if it was (1) reported as having occurred during the trial, (2) reported as part of the estimand definition or (3) mentioned as part of the analysis strategy.

We evaluated whether each of the five standard estimand attributes outlined in the ICH E9(R1) addendum was ‘stated’, ‘inferable’ or ‘not inferable’ using methods similar to those used in other recent reviews of estimands in individually randomised trials.^18,19^ We also evaluated whether the additional considerations specific to CRTs listed in Table 1 were ‘stated’, ‘inferable’ or ‘not inferable’. Rules for determining whether attributes were stated, inferable or not inferable are given in Appendix 5 in the Supplemental Material. Specifically, the handling of individual- and cluster-level intercurrent events attributes would only be applicable if at least one type of cluster-level intercurrent event was reported in the trial.

Based on the evaluation of each attribute, we then evaluated (1) whether we were able to infer all five attributes from the ICH E9(R1) addendum; and (2) whether we could infer the five attributes from the ICH E9(R1) addendum as well as the additional four considerations specific to CRTs.

### Statistical methods

Data were summarised descriptively using frequencies and percentages. All analyses were performed using STATA version 18.

## Results

### Search results and trial characteristics

**Figure 1.**
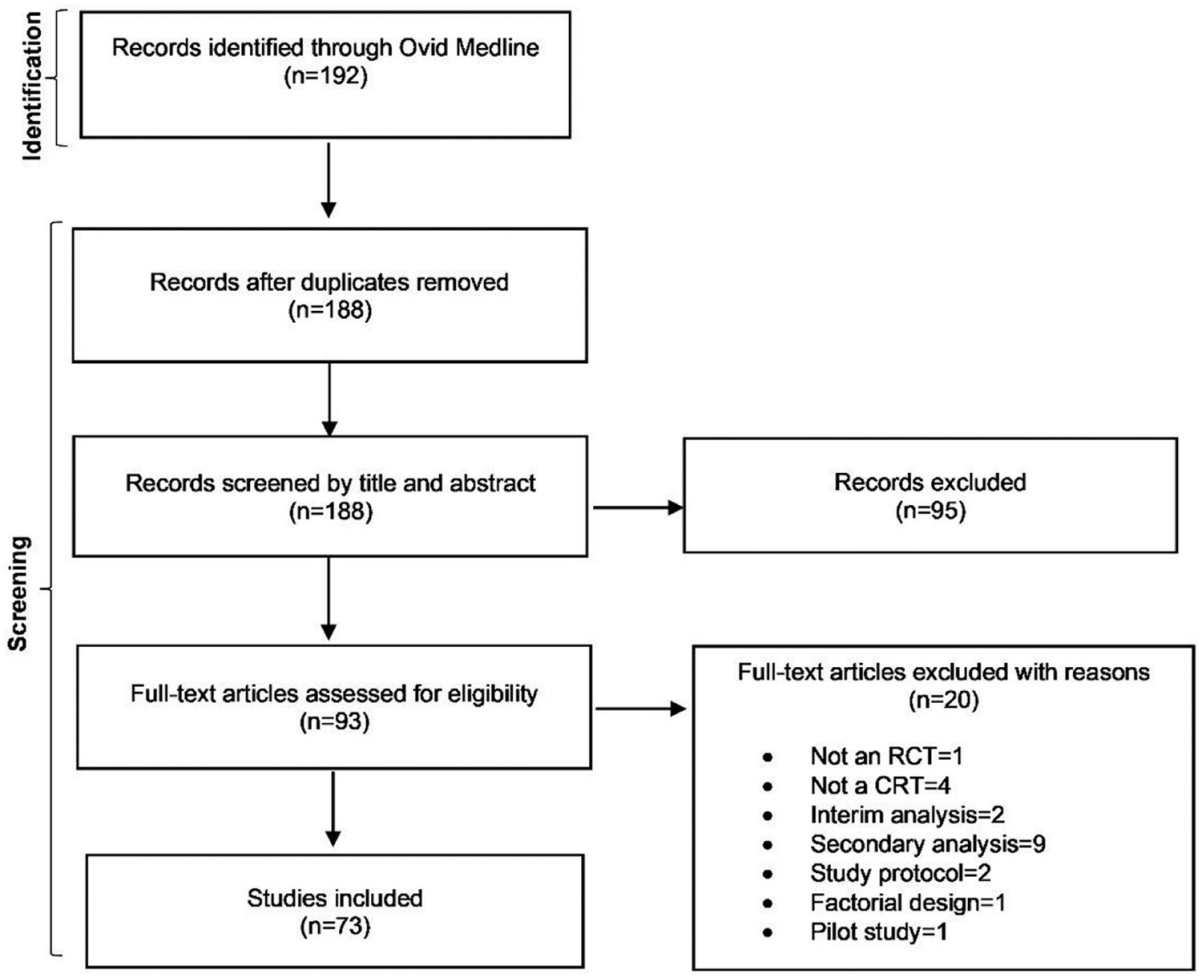
Flow diagram of the search process.

The search identified 192 articles, of which 73 were eligible (Figure 1). The 73 eligible articles were published between 1 October 2023 and 15 January 2024. Most trials had two treatment arms (86%) and used a psychological, behavioural or education intervention (89%). The median sample size was 754 participants (interquartile range (IQR): 101, 3414) and 25 clusters (IQR: 8, 203). The primary outcome was continuous in 36 trials (49%), binary in 25 trials (34%), count in 9 trials (12%) and time-to-event in 3 trials (4%). Further trial characteristics are summarised in Supplemental Table S2 in Appendix 6 in the Supplemental Material.

### Primary estimands

No trials described the estimand for their primary outcome (Table 2). Nevertheless, we were able to infer all five ICH E9(R1) addendum attributes in 36 trials (49%). However, we were only able infer all five ICH E9(R1) attributes and the additional considerations specific to CRTs in 15 trials (21%).

**Table 2.** How well estimands are described in cluster randomised trials.

Characteristic	Trials (N = 73)
Formally tried to describe estimand	0 (0%)
*Standard estimand attributes* ^a^	
Population of patients	
Inferable	46 (63%)
Not inferable	27 (37%)
Treatment conditions	
Inferable	51 (70%)
Not inferable	22 (30%)
Endpoint	
Inferable	73 (100%)
Not inferable	0 (0%)
Summary measure	
Inferable	60 (82%)
Not inferable	13 (18%)
Strategies for handling of individual-level intercurrent events	
Applicable^b^	51 (70%)
Inferable	22/51 (43%)
Not inferable	29/51(57%)
*Additional considerations specific to CRTs* ^a^	
Population of clusters	
Inferable	61 (84%)
Not inferable	12 (16%)
Strategies for handling of cluster-level intercurrent events	
Applicable^c^	33 (45%)
Inferable	26/33 (79%)
Not inferable	7/33 (21%)
How participants are weighted (individual- vs cluster-average effect)	
Inferable	27 (37%)
Not inferable	46 (63%)
Marginal vs cluster-specific effect	
Inferable	70 (96%)
Not inferable	3 (4%)
Overall estimand with standard five attributes^d^	
Inferable	36 (49%)
Not inferable	37 (51%)
Overall estimand including additional considerations^e^	
Inferable	15 (21%)
Not inferable	58 (79%)

a No studies stated any attribute of their estimand.

b Applicable when at least one type of individual-level intercurrent event was reported.

c Applicable when at least one type of cluster-level intercurrent event was reported.

d Population of patients, treatment conditions, endpoint, population-level summary and strategies for handling individual-level intercurrent events.

e Five attributes from ICH E9 (R1) addendum and additional considerations including population of clusters, strategies for handling cluster-level intercurrent events, individual- versus cluster-average effect, marginal versus cluster-specific effect.

A key driver of the ambiguity in the estimand when including the CRT-specific considerations was lack of clarity on whether the effect of interest was the individual-average or cluster-average treatment effect, as we were unable to infer this for 46 trials (63%). We were also unable to infer the population of clusters for 12 trials (16%). The consideration on how cluster-level intercurrent events were handled was applicable in 33 trials (45%) (i.e. at least one type of cluster-level intercurrent event was reported in these trials); among these trials, we were only able to infer the handling strategies for 7 trials (21%) (Table 2).

### Intercurrent events

Sixty-four trials (88%) reported at least one intercurrent event. Fifty-three trials (73%) reported at least one individual-level intercurrent event, and 32 trials (44%) reported at least one cluster-level intercurrent event.

None of the trials in which intercurrent events were applicable clearly stated the strategies they used to handle intercurrent events in their estimand (Table 2). Nevertheless, we were able to infer the strategies for handling individual-level intercurrent events in 22 trials (43%) and cluster-level intercurrent events in 26 trials (79%) (Table 2).

Treatment non-adherence/discontinuation was the most common intercurrent event; it was applicable at the individual level in 38 trials (52%) and at cluster level in 30 trials (41%) (Table 3).

**Table 3.** Whether handling strategies for intercurrent events were inferable or not.

Intercurrent event	Trials n/N (%)
Individual level	
*Treatment non-adherence/discontinuation*	
Applicable	38/73 (52%)
Inferable	24/38 (63%)
Not inferable	14/38 (24%)
*Treatment switching*	
Applicable	4/73 (5%)
Inferable	4/4 (100%)
*Mortality*	
Applicable	19/73 (26%)
Inferable	5/19 (26%)
Not inferable	14/19 (74%)
*Not starting treatment*	
Applicable	19/73 (26%)
Inferable	10/19 (53%)
Not inferable	9/19 (47%)
*Other intercurrent events* ^a^	
Applicable	6/73 (8%)
Inferable	3/6 (50%)
Not inferable	3/6 (50%)
Cluster level	
*Treatment non-adherence/discontinuation*	
Applicable	30/73 (41%)
Inferable	29/30 (97%)
Not inferable	1/30 (3%)
*Not implementing treatment*	
Applicable	15/73 (21%)
Inferable	9/15 (60%)
Not inferable	6/15 (40%)
*Treatment switching*	
Applicable	5/73 (7%)
Inferable	5/5 (100%)
Other intercurrent events^a^	
Applicable	7/73 (10%)
Inferable	7/7 (100%)

a Full list of intercurrent events and the handling strategies can be found in Appendix 6.

In 31 trials (42%), we were able to infer how at least one type of intercurrent event was handled (Table 4). The treatment policy strategy (in which the outcome is of interest regardless of the occurrence of intercurrent event) was the most common strategy and was used to handle all individual-level intercurrent events in 26 trials (84%) and all cluster-level intercurrent events in 30 trials (97%).

**Table 4.** Strategies used to handle intercurrent events.

Item	
By type of intercurrent event	n/N^a^ (%)
Individual level	
*Treatment non-adherence/discontinuation*	
Treatment policy	24/24 (100%)
*Treatment switching*	
Treatment policy	4/4 (4%)
*Mortality*	
Hypothetical	5/5 (100%)
*Not starting treatment*	
Treatment policy	10/10 (100%)
Cluster level	
Not implementing treatment	
Treatment policy	9/9 (100%)
*Treatment switching*	
Treatment policy	5/5 (100%)
*Treatment non-adherence/discontinuation*	
Treatment policy	29/29 (100%)
By handling strategy (where inferable)	n/N^b^ (%)
*Individual level intercurrent events*	
Treatment policy for all IEs	26/31 (84%)
Treatment policy for at least one IE	29/31 (94%)
Hypothetical for at least one IE	5/31 (16%)
Composite for at least one IE	0 (0%)
*Cluster level intercurrent events*	
Treatment policy for all IEs	30/31 (97%)
Treatment policy for at least one IE	30/31 (97%)
Hypothetical for at least one IE	0 (0%)
Composite for at least one IE	1/31 (3%)

a Denominator relates to the number of trials in which the intercurrent event was inferable.

b Denominator relates to the number of trials in which at least one type of intercurrent event’s handling strategy was inferable.

Five trials (16%) used a hypothetical strategy (which considers what the outcome would have been if the intercurrent event had not occurred) to handle at least one individual-level intercurrent event. One trial (3%) used a composite strategy (where a particular outcome value is assigned to those who experience the intercurrent event) to handle at least one cluster-level intercurrent event.

Many trials excluded some clusters (11%) or individuals (30%) from the analysis population (Table 5). This was often done on the basis of clusters/individuals experiencing a specific type of intercurrent events such as not starting treatment or treatment discontinuation. This was a key driver for when the strategies to handle intercurrent events attribute was not inferable, as this way of handling can correspond to different estimands.^18,19^ Reasons why the handling strategy for each type of intercurrent event was inferable or not inferable can be found in Appendix 6 in the Supplemental Material.

**Table 5.** Summary of statistical methods used.

Characteristics	Trials (N = 73)
Statistical method used to analyse primary outcome	
IEEs (independence estimating equations)^a^	6 (8%)
GEEs (exch)	3 (4%)
Mixed-effects models^b^	34 (47%)
Cluster-level summary (marginal)^c^	4 (5%)
Cluster-level summary (cluster-specific)^d^	1 (1%)
Naïve individual-level analyses (ignoring clustering)	15 (21%)
Other^e^	1 (1%)
Unclear^f^	9 (12%)
Used a method that does not make the ‘no informative cluster size’ assumption (e.g. IEEs or cluster summary analysis) as an additional analysis when GEEs/mixed models were used as the main analysis	N = 37
Yes	1 (3%)
No	36 (97%)
Adjusted for cluster size in main analysis model^g^	
Yes	1 (1%)
No	72 (99%)
Reported number of individuals in each cluster	
Yes	3 (4%)
No	70 (96%)
Excluded some clusters from analysis?^h^	
Yes	8 (11%)
No	62 (85%)
Unclear	3 (4%)
Excluded some participants from analysis?^h^	
Yes	22 (30%)
No	45 (62%)
Unclear	6 (8%)

a Defined as any model that used an independence working correlation structure alongside cluster-robust SEs.

b Defined as any hierarchical model with a random intercept for cluster.

c Defined as any analysis that is performed on cluster-level summaries to obtain an overall marginal (population average) treatment effect.

d Defined as any analysis that is performed on cluster-level summaries to obtain a cluster-specific treatment effect.

e A multistate model.

f Denotes any trial in which the model used for the statistical analysis was not reported in sufficient details to understand which model was used.

g The main analysis model included cluster size as a fixed effect. This may be done as an attempt to mitigate against bias from informative cluster size.

h Any participant/cluster that was enrolled but not included in analysis.

### Statistical models

The most common statistical model was the mixed-effect model (n = 34, 47%) (Table 5). IEEs were used in six trials (8%), GEEs (exch) in three (4%) and analysis of cluster-level summaries in five (6%). For nine trials (12%), it was unclear what estimation method was used as insufficient details were reported.

Of the 11 trials (14%) that used IEEs/cluster-level summaries, no trials clearly stated what the target estimand was (i.e. whether the target effect was individual- average or cluster-average), and therefore, it was impossible to infer whether these methods aligned with the trial’s objective.

Over half of trials (n = 37, 51%) used GEEs (exch) or mixed-effect models, which rely on the assumption that there is no informative cluster size in order to be unbiased. However, only one trial (3%) performed an additional analysis using a method that does not rely on the assumption of non-informative cluster size (e.g. using IEEs/cluster-level summaries) as a sensitivity analysis. Only three trials (4%) reported the size of each cluster, making it difficult to assess whether informative cluster size was a possible concern.

## Discussion

### Principal findings

Despite publication of the ICH E9(R1) addendum in 2019, we found no evidence of uptake of estimands in our review of CRTs published between October 2023 and January 2024. Among the 73 trials we reviewed, no trial attempted to describe the estimand for their primary outcome.

This lack of uptake of estimands had major implications for our ability to decipher trial objectives. In over half of trials (51%), we could not infer which estimand was being targeted by the trial’s estimator based on the standard five attributes outlined in the ICH E9(R1) addendum; we were unable to infer all five ICH E9(R1) attributes and additional considerations specific to CRTs for 79% of trials.

In addition to creating ambiguity around trial objectives, failure to specify the estimand also made it difficult to evaluate the appropriateness of the chosen statistical estimator, as well as the assumptions they made. For instance, 14% of trials used IEEs/cluster-level summary analyses without a clear indication of the target estimand, so it was impossible for us to determine whether the weighting scheme used was appropriate for trial objectives. Similarly, although 51% of trials used mixed-effects models or GEEs (exch), almost none used sensitivity analyses to evaluate robustness of these results to departure from the ‘no informative cluster size’ assumption, nor reported the size of each cluster to allow readers to infer whether informative cluster size was a potential concern.

It was concerning to us how some trials handled intercurrent events in their analysis. For instance, 11% of trials excluded some clusters from the analysis; however, without further explanation or justification of this approach, it is difficult to understand which estimand is being targeted, or whether such an approach is justified. Furthermore, based on the statistical methods used, we identified that 16% of trials for which the strategy was inferable used a hypothetical strategy to handle individuals who died (i.e. estimated what the treatment effect would have been had no patients died). However, it is not clear whether investigators intended to implement this strategy, which has been criticised,^18,19^ or whether this was a simple happenstance based on the choice of analytical model.

### Implications of findings

Recent reviews have been undertaken to explore the use of estimands in individually randomised trials.^18,19^ These reviews found low levels of use; however, they included trials published in 2020, just a year after the ICH E9(R1) addendum was published, which limits the comparability to this review. Interestingly, the primary estimand was inferable in 46% of the published individually randomised trials in the review by Cro et al.,^19^ whereas the primary estimand (including both the ICH E9(R1) attributes and additional CRT considerations) was inferable in only 21% of the CRTs in our review. This provides evidence that additional guidance on defining estimands in CRTs is needed.

A main driver in the ambiguity around target estimands was lack of clarity around some of the CRT-specific considerations which are not explicitly described in the ICH E9(R1) addendum. For instance, we could not infer whether investigators were interested in individual- or cluster-average effects for 63% of trials. This is because the dominant method to analyse CRTs is based on mixed-effects models or GEEs (exch), for which the implicit weighting mechanism corresponds to neither the individual-average (where individuals receiving equal weight) or the cluster-average (where clusters receiving equal weight) effect. Instead, these two estimators weight clusters by their inverse-variance, which is a function of the intraclass correlation coefficient and the cluster size.^9,17^

Of note, it was only recently that some of these additional considerations for estimands in CRTs have been highlighted in the literature (e.g. two papers outlining the distinction between individual- vs cluster-average estimands were published by Wang et al.^17^ and Kahan et al.^9^). Many articles in this review will have been designed before then, which may explain why none tried to address these additional considerations. Nevertheless, this highlights that (1) the adoption of estimands in CRTs is low, despite the ICH E9(R1) addendum having been published in 2019; and (2) defining estimands according to the framework set out in the ICH E9(R1) addendum is not sufficient to clearly define the research question of interest in CRTs. This motivates the need for specific guidance for defining estimands in CRTs, which should include the considerations that are specific to CRTs, such as how individuals and clusters are weighted, and how cluster-level intercurrent events are handled.

This work also highlights the need for methods to increase uptake of estimands in CRTs. The CONSORT extension for CRTs was published in 2012, prior to publication of the ICH E9(R1) addendum. In any future updates of the CONSORT extension for CRTs, it would be useful to consider estimands as a potential reporting item, as this would help ensure that reports of CRTs clearly articulate their estimand, thereby allowing readers to better understand trial objectives, as well as to facilitate critical appraisal of statistical methods.

### Limitations and strengths

A limitation of the study is that only one database (MEDLINE) was searched while multiple databases are recommended for systematic reviews. However, MEDLINE has good coverage of medical journals that are likely to publish relevant CRTs,^20^ and our aim was to obtain a broad snapshot of current practice around the use of estimands in CRTs rather than comprehensively evaluate every single published CRT. Thus, the use of a single database was deemed sufficient for our objective. No formal sample size calculation was performed for this review. However, our sample of n = 73 has provided clear evidence that estimands are not being used in CRTs and identified clear areas for improvement. It is unlikely that a larger sample size would alter this conclusion.

In addition, we did not consider methods used to address missing outcome data when trying to infer the estimand. This would be a useful area of future research, to evaluate how the way missing data are handled and whether that is in line with the target estimand in CRTs.

The study had several strengths, including piloting of the data extraction form, as well as data extraction and checking by two independent statisticians to help minimise extraction errors.

## Conclusion

The uptake of estimands in published CRT articles is low, making it difficult to ascertain which questions were being investigated or whether statistical estimators were appropriate for those questions. This highlights an urgent need to develop guidelines on defining estimands that cover unique aspects of CRTs to ensure clarity on research questions in these trials, as well as to consider the inclusion of estimands in any update to reporting guidelines for CRTs such as the CONSORT extension.

## Supplemental Material

sj-docx-1-ctj-10.1177_17407745251415538 – Supplemental material for Use of estimands in cluster randomised trials Supplemental material, sj-docx-1-ctj-10.1177_17407745251415538 for Use of estimands in cluster randomised trials by Dongquan Bi, Andrew Copas and Brennan C Kahan in Clinical Trials
